# Development and Implementation of ‘Just Right’ Physical Behavior in Industrial Work Based on the Goldilocks Work Principle—A Feasibility Study

**DOI:** 10.3390/ijerph18094707

**Published:** 2021-04-28

**Authors:** Anders Fritz Lerche, Svend Erik Mathiassen, Charlotte Lund Rasmussen, Leon Straker, Karen Søgaard, Andreas Holtermann

**Affiliations:** 1 The National Research Centre for the Working Environment, 2100 Copenhagen, Denmark; clr@nfa.dk (C.L.R.); aho@nfa.dk (A.H.); 2 Department of Sports Science and Clinical Biomechanics, University of Southern Denmark, 5230 Odense M, Denmark; ksogaard@health.sdu.dk; 3 Centre for Musculoskeletal Research, Department of Occupational and Public Health Sciences, University of Gävle, 801 76 Gävle, Sweden; SvendErik.Mathiassen@hig.se; 4 School of Allied Health, Curtin University, Perth, WA 6845, Australia; L.Straker@curtin.edu.au; 5 Department of Clinical Research, University of Southern Denmark, 5230 Odense M, Denmark

**Keywords:** physical behavior, health, workplace, intervention, Goldilocks Work Principle

## Abstract

The Goldilocks Work Principle expresses that productive work should be redesigned to comprise physical behaviors of different intensities in a composition promoting workers’ health and fitness. This study is the first to assess the feasibility of redesigning work in an industrial setting according to the Goldilocks Work Principle. We recruited workers (*n* = 20) from a brewery in Denmark, and we conducted a participatory 16-week intervention including a workshop and two consultations. The workshop aimed to support the workers in modifying their work, while the consultations assisted the eventual implementation. Feasibility was evaluated as per three aspects: (1) developing modifications of work, (2) implementing these modifications, and (3) changing physical behavior and self-reported fatigue, pain and energy. The three aspects were addressed through records completed by the workers, measurements of workers’ physical behavior and intensity during ‘control’ workdays (i.e., usual work) and ‘intervention’ workdays (i.e., modified work), and self-reported fatigue, pain and energy level following both types of workday. Five modifications to work were developed, and three of these five modifications were implemented. To some extent, physical behavior and intensity changed as intended during ‘intervention’ workdays compared to ‘control’ workdays. Workers were also less fatigued, had less pain, and had more energy after ‘intervention’ workdays. These results suggest that it is feasible to develop and implement modified work based on the Goldilocks Work Principle among industrial workers. However, we also identified several barriers to the implementation of such modifications.

## 1. Introduction

Industrial workers often have work tasks leading to considerable physical demands for several hours every workday [1]. Measurements of physical behavior using wearable sensors show that industrial workers can spend a large fraction of their workday standing (>50%), while the remaining time is spent sitting or active (e.g., walking, stair climbing) [2]. This physical behavior during work does not appear to promote the health of the workers [3]. On the contrary, workers exhibit a high prevalence of musculoskeletal disorders (MSD), poor general health and low fitness levels [2].

This suggests that occupational physical activity (OPA) does not lead to the same health benefits as leisure time physical activity [3]; this has been termed the “physical activity paradox” [4]. High levels of OPA have been shown to be associated with a higher risk of work-related musculoskeletal disorders [5], elevated 24 h heart rate and blood pressure, as well as an increased risk of sickness absence, cardiovascular disease and all-cause mortality [4,6,7]. The negative health effects may be explained by OPA not offering the same health-promoting elements as leisure time physical activity, such as sufficient variation between physical behaviors, including both active periods at an intensity sufficient to promote fitness and sufficient periods with recovery [8]. Industrial work is often performed standing for extended periods of time, which may increase pain and discomfort in the lower back and lower extremities [9,10] and be associated with negative effects on cardiovascular health [11,12]. Furthermore, when industrial workers are physically active during work, the intensity does not seem to be sufficient to promote cardiorespiratory fitness [3]. Thus, for industrial workers the distribution of physical behaviors with different intensities across the workday may not be ‘just right’ for promotion of health and fitness.

A promising approach for improving this situation is the Goldilocks Work Principle, saying that productive work should be redesigned to promote health and fitness by shifting workers’ physical behaviors towards a ‘just right’ distribution, including periods of sufficient intensity to promote fitness [13,14]. For example, industrial workers who are standing most of their workday may benefit from having more periods being active, to promote fitness, but even more periods sitting, to allow recovery [15]. As a basis for designing a modification of any specific work situation according to the Goldilocks Work Principle, information is needed about the workers’ health and fitness status, the exposures associated with the available work tasks, and the current organization of these tasks [13,14]. The next step recommended is to redesign the productive work through a participative process with the workplace. This redesign process has been tested among childcare workers with promising results [16]. In that study a common work task (i.e., pedagogical games) was redesigned according to the Goldilocks Work Principle in order to elicit periods with high intensity among the childcare workers, while it would still be pedagogically relevant for the children [16]. The aim of increasing time with high intensity was determined after identifying those physical behavior changes in childcare work that would likely have the best potential to be implemented, while also improving health and fitness of the childcare workers. Since industrial workers appear to have another health profile and other physical behaviors at work than childcare workers, and since industrial work is organized, planned and performed in a different manner, more information is needed regarding the feasibility of redesigning work to fit an industrial setting.

Therefore, the aim of this study was to investigate the feasibility of (1) developing modifications to industrial work according to the Goldilocks Work Principle, (2) implementing these modifications; (3) changing workers’ physical behaviors towards a ‘just right’ distribution, including sufficient sitting to gain recovery and periods of a sufficiently high physical intensity to improve fitness; resulting in fatigue, pain and energy following work changing in a positive direction.

## 2. Materials and Methods

### 2.1. Data Protection and Ethical Approval

The National Research Centre for the Working Environment has an institutional agreement with the Danish Data Protection Agency about procedures to treat confidential data (journal number 2015-41-4232), e.g., by securing data at a protected drive with limited access, and by anonymizing all individual data.

The Danish National Committee on Biomedical Research Ethics (the local ethical committee of Frederiksberg and Copenhagen) has evaluated a description of the study and concluded that, according to Danish law as defined in Committee Act § 2 and § 1, the intervention should not be further reported to the local ethics committee (Ref number: H-18041423).

The reporting follows the Consolidated Standards of Reporting Trials (CONSORT) 2010 statement extension to randomized controlled pilot and feasibility trials [17]. Additionally, the intervention description follows the Template for Intervention Description and Replication (TIDieR) checklist [18].

### 2.2. Participants

Industrial workers (*n* = 26) were recruited from a brewing company in Denmark. The study was announced at a quarterly meeting with both senior management and workers present. For logistical reasons we included workers exclusively from one department of the work production. This department was selected based on discussions with the management. The priority was to identify a department with interest in participation and with a potential to benefit from this type of intervention. The department consisted of three teams, with two teams weekly alternating between day and evening shifts, and one team exclusively doing night shifts. The main task in the department was to handle products (e.g., relocate boxes with bottles, assemble pallets and attach labels to bottles) that were not suitable for processing by the company’s automated robotics system. We invited workers from the day and evening teams to participate in this study.

Exclusion criteria were: pregnancy, adhesive tape allergy, fever on the day of baseline measurements, and use of a pacemaker. All participants provided written informed consent prior to participating in the study.

### 2.3. The Four Procedural Steps in Developing Goldilocks Work Interventions

**Figure 1:**
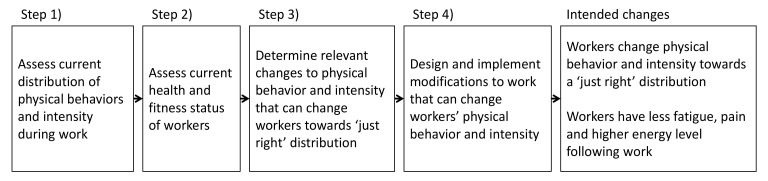
Four steps to change workers physical behavior and intensity towards a ‘just right’ distribution according to the Goldilocks Work Principle, and the intended changes.

To guide the process of designing and implementing the workplace intervention, we used the recommended 4-step procedure shown in Figure 1 [14]. We initially established management support, and formed a local workplace group (LWG) consisting of the team leader, a health and safety specialist and the work environment representative (WER), for collaboration and consultancy throughout the intervention.

In step 1, we discussed, with the LWG, the potential for modifying work in the present population of workers. Subsequently, we identified all work tasks and identified those occurring the most. We then assessed the physical behaviors associated with performing these main tasks, using field observations and dialogues with workers and the LWG. Additionally, the workers’ physical behaviors and intensity during five ‘control’ working days were measured using wearable sensors (detailed in Appendix A, Table A1).

In step 2, we assessed the health status of workers in the department by collecting information about age, smoking status, body mass index (BMI), blood pressure and fat percentage, perceived physical exertion during work, and self-reported physical activity level during work and leisure.

In step 3, the information from Step 1 and 2 provided a basis for assessing desirable changes in physical behavior which would lead towards a ‘just right’ distribution of behaviors, and to a sufficient occurrence of periods at high intensity.

In step 4, we invited all workers in the department and the LWG to participate in the intervention, including being involved in the process of modifying their productive work and subsequently implement these modifications with the aim to achieve the intended goals. These steps are described in detail in Appendix B.

### 2.4. Operational Definition of the ‘Just Right’ Distribution

In 2020, the World Health Organization (WHO) updated their guidelines on physical activity and sedentary behavior for adults to recommend between 150 and 300 min per week of physical activity at moderate intensity or 75 to 150 min at high intensity, while also reducing time in sedentary behavior [19,20]. Briefly, these recommendations are based mainly on a well-established positive dose-response relationship between time spent in leisure time physical activity and health [21]. However, implementing these recommendations in the context of productive industrial work may not be feasible or desirable. For example, workers with high amounts of occupational physical activity have been shown to have higher risk of getting cardiovascular diseases [22], whereas occupational sitting among industrial workers has been shown to have a beneficial effect on low back pain [23] and long-term sickness absence due to musculoskeletal pain [24]. Thus, following the WHO guidelines of ‘move more, sit less’ does not seem to be the most suitable approach among workers in this occupational sector [25,26].

To our knowledge, the ‘just right’ distribution of physical behaviors during work that would improve health and fitness is unknown. The evidence of physical variation being a protective factor against musculoskeletal disorders in working life, as suggested mainly for jobs characterized by continuous and/or repetitive operations, is weak [27]. A systematic review found inconsistent evidence supporting job rotation as a strategy for preventing musculoskeletal complaints [28], while another review found some positive effects of increasing physical variation [29]. The primary explanation for the weak evidence regarding physical variation is suggested to be a lack of standardized methods for assessing variation, and few high-quality studies assessing both exposure variation and musculoskeletal health outcomes [27]. Hence, there is a need to establish which occupations that could benefit from interventions addressing physical variation, and what an optimal variation might be in those cases [27]. A balanced workday with good variation [27], where the worker does not spend most of the time in one behavior (e.g., sitting, standing, or being physically active) working at about the same intensity (e.g., low, moderate or high intensity) could likely contribute to reduce fatigue, prevent musculoskeletal disorders [5], decrease long-term sickness absence [30] and improve workers’ general health [14]. Furthermore, including high-intensity physical activity to a sufficient extent, complementing physical activity performed at lower intensities [15,31], could be an effective way of enhancing workers’ health and cardiorespiratory fitness [19,20,31,32].

In order to assess the feasibility of changing physical behaviors based on the Goldilocks Work Principle, therefore, we decided on an operational definition of a ‘just right’ distribution of activities during a workday. We described it as follows: (1) to be characterized by one third of the time at work spent in each of these behaviors; sitting, standing and being active, and (2) to contain at least 10 min of high physical intensity, operationalized as activity associated with a heart rate equal to, or larger than 60% of the heart rate reserve (HRR). We considered these target behaviors to be feasible as part of the productive work, as well as sufficient for having a positive impact on workers’ health and cardiorespiratory fitness.

### 2.5. Workplace Intervention

**Figure 2:**
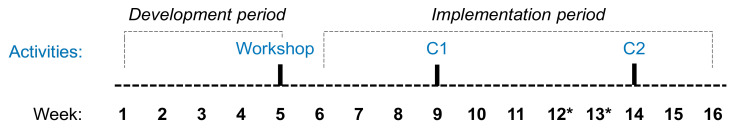
Timeline of the development and implementation of the intervention. Blue color indicates the intervention activities. The first and second dashed brackets indicate the periods where modifications to work were developed and implemented, respectively. C1: Consultation 1, C2: Consultation 2. * indicates weeks excluded due to holidays.

All workers in the department (i.e., day and evening shift) participated in a 16-week intervention (November 2019 to January 2020; excluding two weeks due to end of year holidays). Figure 2 presents the intervention timeline.

The development period consisted of an ongoing collaboration with the LWG to identify desirable changes to current work. Furthermore, two workshops were conducted, for the day and evening shift workers, respectively. The LWG were present at both workshops. We initiated the workshops by explaining the Goldilocks Work Principle and presenting a summary of their own baseline measurements. Then, the workers were split into groups, with a request to design modifications to their work. Each idea for modification was assessed by all workshop participants in terms of five criteria: (1) would it contribute to changing physical behavior and/or intensity towards a ‘just right’ distribution? (2) Is it a part of productive work? (3) Are there facilitating factors supporting implementation of this modification (e.g., fun to do)? (4) Are there barriers to the implementation of this modification (e.g., expensive, not practical)? (5) How would the modification affect overall productivity? All modifications were scored in each category as either positive, neutral, or negative. Based on this assessment, the LWG selected the modifications considered to have the best potential to be successfully implemented. The workshop contents are detailed in Appendix C (Table A2).

The implementation period included two consultations (C1 and C2) conducted by the researchers with the purpose to assist the LWG in the implementation of modifications. In the consultations, the LWG used a checklist to evaluate whether each of the modifications was performed by the workers. If a modification had not been performed, the LWG initiated a discussion of the reasons why, and how to facilitate a future implementation.

### 2.6. Data Collection

**Figure 3:**
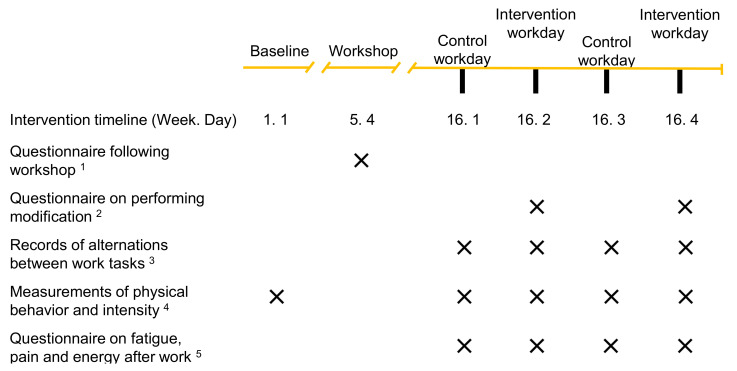
Measurements during the implementation. ^1^ Workers filled out a questionnaire following the workshop. ^2^ All workers answered a questionnaire addressing whether they had performed each of the work modifications as intended. If they had not, they could state the reason. ^3^ After work, workers noted the number of alternations between work tasks. ^4^ Workers wore sensors measuring physical behaviors and heart rate during work. ^5^ Workers answered a questionnaire on perceived fatigue, pain and energy wrt. performing physical activity after work.

At baseline (cf. Figure 3), we collected self-reported information on age, sex, current smoking habits, job title, current length of service, working hours per week, self-rated productivity [33], perceived physical exertion during work (Borg’s CR10 scale; [34]) and highest perceived pain intensity within the last week [35]. Additionally, workers answered the Nordic Physical Activity Questionnaire-short [36] and reported time spent in moderate to vigorous physical activity (MVPA), including time specifically spent with vigorous physical activity (VPA). Furthermore, we measured body height [cm], weight [kg], fat percentage (BC-418 MA body composition analyzer; Tanita, Tokyo, Japan), resting blood pressure (Omron M3 r Omron M6 Comfort; Omron Corporation, Kyoto, Japan), and we calculated the BMI (weight [kg]/(height squared [m^2^])).

At the end of the two workshops, the participating workers’ opinions were collected using a questionnaire (Figure 3), which addressed the extent to which the respondent agreed to statements about their motivation, the content of the workshop and management support, with response alternatives: (1) not at all; (2) to a low degree; (3) to some degree; (4) to a high degree; (5) to a very high degree. Answers were collapsed into three categories, i.e., “to a low degree” (1 and 2), “somewhat” (3) and “to a high degree” (4 and 5). Furthermore, workers were asked to rate the performance of the workshop leader from 0 (worst imaginable performance) to 5 (best possible performance). The team leader and WER were present at both workshops, but only filled out the questionnaire with the day shift. Answers from both workshops were pooled. The contents of the questionnaire are detailed in Appendix D (Table A3).

At follow up, we measured (1) to what extent the modifications had been implemented as planned, (2) to what extent workers’ physical behavior and intensity had changed as intended, and (3) to what extent the implemented modifications had led to short-term positive changes in workers’ health and wellbeing (fatigue, pain and energy following work). The LWG informed workers when the follow up measurements would be performed, and encouraged workers to participate and wear sensors for recording physical behavior and intensity. Measurements were performed during a 4-day period with a randomized order of two ‘control’ workdays (tasks performed as usual) and two ‘intervention’ workdays (modified tasks).

#### 2.6.1. Compliance

To examine the workers’ compliance to each of the five modifications, they were asked (yes/no) on day 2 and 4 whether they had performed their work according to the plan (Figure 3). On answering ‘no’, the worker was requested to explain why.

Furthermore, at the end of both ‘control’ and ‘intervention’ workdays, the worker noted the total number of work tasks performed during the day, as well as the number of alternations between tasks with different physical behaviors. Both variables reflect the frequency aspect of variation [27]. This report was reviewed post-hoc by the researchers, so that only alternations that did, indeed, occur between sufficiently different tasks were counted.

#### 2.6.2. Physical Behavior and Intensity

We used a protocol for measuring physical behavior and intensity similar to that in a previous study among childcare workers [16]. An AX3 accelerometer (3-Axis Logging Accelerometer; Axivity Ltd., Newcastle upon Tyne, UK) was mounted using adhesive tape (Hair-Set double-sided adhesive tape; 3 M Company, Maplewood, MN, USA) on the worker’s right thigh at the most muscular part of the quadriceps femoris, midway on the line between the anterior inferior iliac spine and the top of the patella. A Firstbeat Bodyguard 2 heart rate monitors (Firstbeat Technologies Ltd., Jyväskylä, Finland) was placed using Ag/AgCl pre-gelled electrodes below the right clavicle and at the left rib cage. Workers were asked to wear the equipment around the clock for four days, and were instructed on how to change the adhesive tape if necessary. After four days, data were downloaded using the manufacturer’s software (Firstbeat Uploader Version 3.1.2.0; Firstbeat Technologies Ltd., and OMGUI, Configuration and Analysis Tool) and further processed for evaluation of beat errors and physical behaviors using a MATLAB based software (Acti4; The National Research Centre for the Working Environment, Copenhagen, Denmark) [37,38]. A workday was excluded from further analysis if the heart rate recording was contaminated by beat errors ≥50% of the time [2,37,39], or if accelerometer recordings were only available for less than 4 h.

Physical behaviors were classified using the Acti4 software [38] into time spent sitting or lying (termed sitting below), standing (consisting of time spent standing still, or standing with slight movement), and active (consisting of time spent walking, running, stair climbing or cycling). Periods >60 min without movement were regarded as non-wear time or sleep.

In order to classify the intensity of physical activity as low, moderate or high, we estimated the heart rate reserve (HRR), i.e., the difference between the maximal heart rate, estimated according to Tanaka et al. [40], and resting heart rate. Resting heart rate was defined as the lowest recorded heart rate during the first night’s sleep [2,39]. Low intensity was defined as time at <25%HRR [41]. Moderate intensity was defined to occur at ≥25 %HRR, but <60%HRR. High intensity was expressed in terms of time ≥60%HRR, since intensities at and above this threshold have been shown to be effective in improving cardiovascular fitness [31,42].

During all measurement days, the workers noted in a diary at what time they, (1) woke up, (2) arrived at work, (3) left work, (4) went to sleep, and (5) if any of the wearable sensors were detached. On basis of the diaries, the continuous timeline of heart rate and physical behavior data was partitioned into periods of, (1) ‘control’ work, (2) ‘intervention’ work, (3) leisure time awake, and (4) sleep. Workers with data on both ‘control’ and ‘intervention’ workdays were included in further analyses, and their data on ‘control’ and ‘intervention’ work were summarized in terms of a mean value for ‘control’ and ‘intervention’ workdays.

#### 2.6.3. Fatigue, Pain and Energy

At the end of each ‘control’ or ‘intervention’ workday (Figure 3), workers rated fatigue and pain in the neck and shoulders, lower back and legs and knees on a scale from 0–10 [35]. Furthermore, they were asked to rate (also from 0 to 10) their energy to perform 30 min of physical activity after work, as either walking, running or sporting [34]. Ratings for ‘control’ and ‘intervention’ workdays were summarized by mean values.

### 2.7. Effects of Modifications

Due to the small sample size and exploratory nature of this feasibility study, we used only descriptive statistics, and did not compare ‘control’ and ‘intervention’ workdays using more advanced statistical procedures. Group means with standard deviation (SD) between workers were used to summarize data on worker characteristics, physical behaviors, heart rate, and ratings in questions on fatigue, pain and energy. All categorical data are presented as N (number of workers), and percentages of the total population.

## 3. Results

### 3.1. Participant Flow

**Figure 4:**
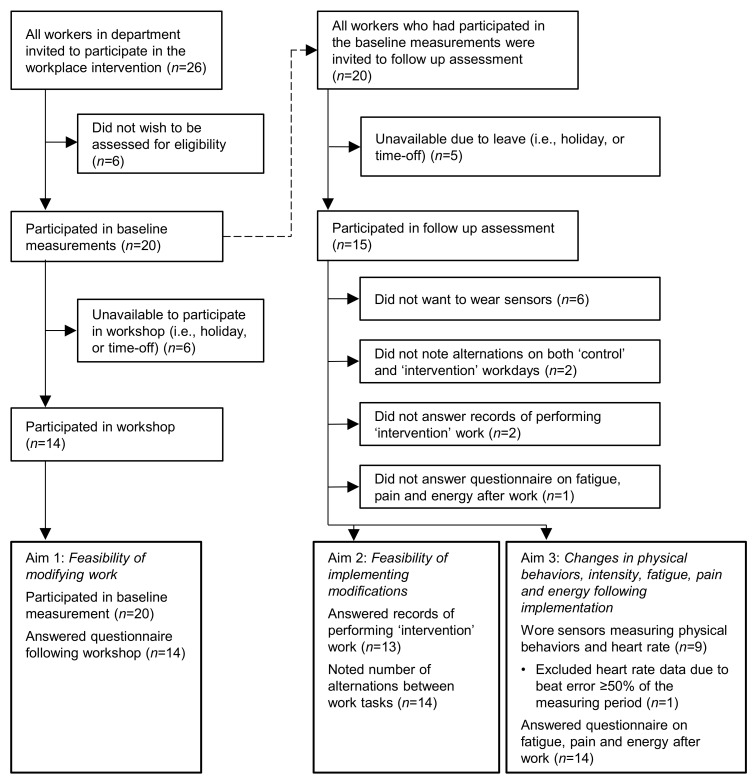
Participant flow, illustrating the amount of data available for answering Aims 1, 2 and 3.

Out of 26 eligible workers, we collected baseline data on 20 (Figure 4). All 14 workers attending the workshops, as well as the team leader and WER, provided data on their opinions regarding the workshop. Thirteen out of the 15 workers participating in follow-up measurements gave information on their ability to perform the planned modifications, and 14 reported the number of alternations between work tasks with different physical behaviors. Nine workers had data on physical behavior using accelerometers, and eight had heart rate data. Six workers did not wish to wear the sensors, the primary reason being that they were concerned that management might use the eventual data against them. This concern appeared despite several efforts made by the research team to assure workers that several measures were taken to secure that such a scenario could not occur. Fourteen reported their perceived fatigue, pain and energy level on both ‘control’ and ‘intervention’ workdays.

### 3.2. Worker Characteristics

Workers participating at baseline (*n* = 20) were predominantly male (Table 1). Workers claimed a high productivity and reported a ‘strong’ physical exertion during work on the Borg CR10 scale. They rated pain within the last week in the neck/shoulder, lower back and knees to be of mild to moderate intensity. Twenty percent of the workers were obese and 30% had hypertension.

### 3.3. Development of ‘Just Right’ Work Modifications

**Figure 5:**
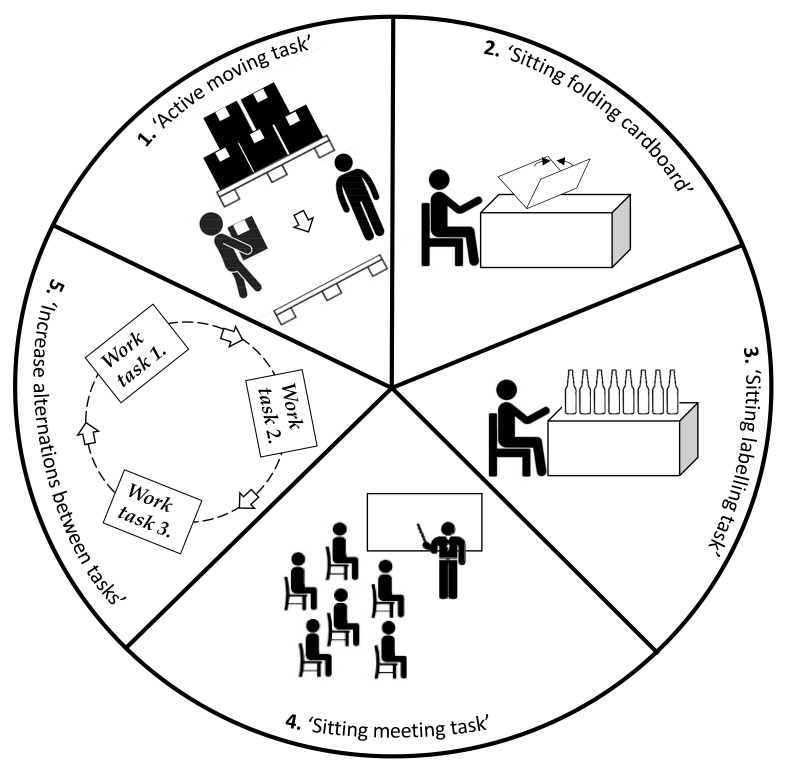
Illustrations of the five modifications developed at the workshop.

Five modifications of work were developed at the workshops aiming at designing a ‘just right’ distribution of physical behaviors, as well as getting more time at a high intensity during productive work (Figure 5).

Modification 1; ‘Active moving task’. The task consisted in relocating and labeling boxes with bottles from one pallet to another. This task was originally performed by a single worker at a slow, steady pace. The work task was modified to engage two workers alternating between (1) moving boxes with bottles in a rapid pace and (2) labeling the boxes just moved. The intention of this modification was to increase time being physically active at a high intensity.

Modification 2; ‘Sitting folding cardboard’. The task consisted in assembling a quarter pallet of bottles. This was originally done by folding a cardboard on top of a quarter pallet and placing bottles there in layers. The work task was modified by creating a new task consisting in folding cardboards supplying workers placing bottles on pallets. The new cardboard folding task could be performed sitting, and workers were encouraged to do so. The purpose of this modification was to increase time sitting.

Modification 3; ‘Sitting labelling task’. This work task consisted in labeling bottles. This was originally done in a standing position with little movement. The work task was modified by making a chair available and encouraging the workers to sit while labeling the bottles. The purpose of this modification was to increase periods of sitting.

Modification 4; ‘Sitting meeting task’. The task included planning and distributing tasks among workers. The meeting was conducted at the beginning of each workday, with participants standing next to a blackboard. This task was modified by placing out pallets for sitting and encouraging workers to use them while meeting. The purpose of this modification was to increase time with sitting.

Modification 5; ‘Increase alternations between tasks’. The intention of this modification was to reorganize work so that workers alternated twice every workday between work tasks 1, 2 and 3 above, in their modified form, as opposed to the original rotation of only one alternation per day between the three tasks in their original form. The purpose of this modification was to increase variation in physical behavior and increase time being active.

### 3.4. Workers’ Opinions of the Workshop

Workers (*n* = 14) were in general motivated to participate, felt they benefited from the content of the workshop, and felt that management supported the intervention. All questions and responses are available in Appendix D (Table A3).

### 3.5. Compliance

**Figure 6:**
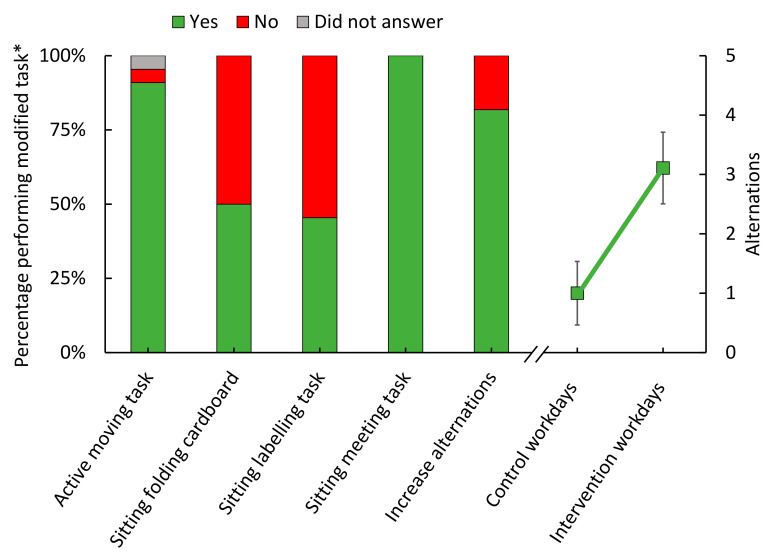
Answers to whether modifications were performed as planned during ‘intervention’ workdays (*n* = 13), and the number of alternations between work tasks on ‘control’ and ‘intervention’ workdays (*n* = 14). The left y-axis represents the percentage of answers ‘yes’, ‘no’ or ‘did not answer’. The right y-axis represents the number of alternations between work tasks with different physical behaviors. Green squares indicate group mean and error bars represent standard deviation (SD) between workers. * Nine workers gave answers following both ‘intervention’ workdays (i.e., 18 answers), and four workers provided answers only on one workday (i.e., 4 answers), adding up to 22 answers (i.e., 100%).

Nine of the 13 workers answering the questionnaire on whether modifications were, in fact, performed during ‘intervention’ workdays answered on both those days (i.e., 18 workdays), while four workers answered on only one (i.e., 4 workdays). Workers reported that they were able to perform the ‘active moving task’ (91%), ‘sitting meeting task’ (100%) and ‘increase alternations’ (82%) modifications as planned on the majority of the workdays (Figure 6). A considerable proportion of the workers reported that they were not able to perform ‘sitting folding cardboard’ (50%) and ‘sitting labelling task’ (55%) as planned (Figure 6). The primary reason for not performing ‘sitting folding cardboard’ as planned was that a colleague had already performed the task, and that it was, therefore, no longer needed. The primary reason for not performing ‘sitting labelling task’ as planned was that the chair was uncomfortable.

Thirteen workers answered regarding the number of alternations between work tasks during ‘control’ and ‘intervention’ workdays. Workers typically alternated once during ‘control’ workdays, increasing to three times on ‘intervention’ workdays (Figure 6). This shows that variation increased in terms of the frequency of alternations between work tasks with different physical behavior.

### 3.6. Physical Behaviors and Intensity

**Figure 7:**
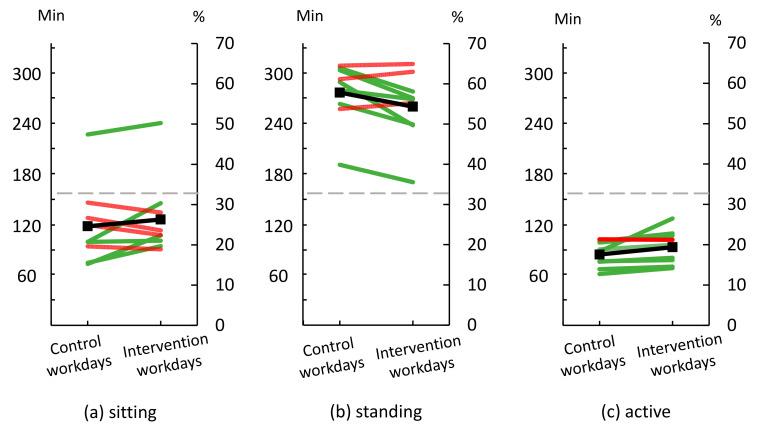
Time spent in different physical behaviors, i.e., (**a**) sitting, (**b**) standing and (**c**) active during ‘control’ and ‘intervention’ workdays (*n* = 9). ‘Control’ workdays consisted of performing work as usual, and ‘intervention’ workdays consisted of performing modified work. The right y-axis shows percentage time spent in each behavior during work; the left y-axis shows the corresponding number of minutes. Grey dashed lines indicate proportions of behaviors in a ‘just right’, balanced distribution. Green lines illustrate workers changing towards this ‘just right’ distribution. Red lines illustrate workers who move away from this distribution. Black lines show group means.

Data on time spent sitting, standing and being active were available from nine workers who wore accelerometers on both ‘control’ and ‘intervention’ workdays (Figure 7). On ‘control’ workdays, workers were, on average, sitting for 24.6% of the time (SD 9.2), standing for 57.8% of the time (SD 7.3), and active for 17.6% of the time (SD 3.0). With a workday of 480 min, these proportions would correspond to 118 min, 278 min, and 84 min, respectively. During the ‘intervention’ workdays, workers were sitting for 26.3% of the time (SD 9.2), standing for 54.3% of the time (SD 8.2), and active for 19.4% of the time (SD 4.0). With a workday of 480 min, these proportions would correspond to 126 min, 261 min, and 93 min, respectively.

**Figure 8:**
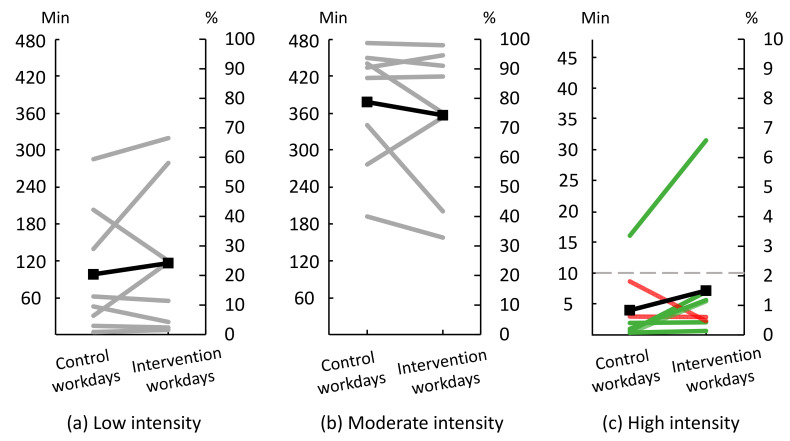
Time spent at (**a**) low intensity, i.e., <25%HRR (heart rate reserve), (**b**) moderate intensity, i.e., ≥25–<60%HRR, and (**c**) high intensity, i.e., ≥60%HRR during ‘control’ and ‘intervention’ workdays (*n* = 8). ‘Control’ workdays consisted of performing work as usual; ‘intervention’ workdays consisted of performing modified work. The right y-axis shows the percentage time spent in each intensity during work; the left y-axis shows the corresponding number of minutes. Grey lines illustrate individual workers’ results for low and moderate intensity. In the high intensity panel, green lines illustrate workers already having a ‘just right’ distribution, or changing towards it. Red lines illustrate workers not having a ‘just right’ distribution, and moving away from it. Black lines show group mean. The grey dashed line indicate the ‘just right’ amount of 10 min of high intensity.

Data on time spent at low intensity, moderate intensity and high intensity were available from eight workers who wore heart rate sensors on both ‘control’ and ‘intervention’ workdays (Figure 8). On ‘control’ workdays workers spent on average 20.4% of the time (SD 19.8) in low intensity, 78.8% of the time (SD 19.4) in moderate intensity, and 0.8% of the time (SD 1.1) in high intensity. With a workday of 480 min, these proportions correspond to 98 min, 378 min, and 4 min, respectively. On ‘intervention’ workdays workers spent 24.2% of the time (SD 23.7) in low intensity, 74.3% of the time (SD 22.9) in moderate intensity, and 1.5% of the time (SD 2.0) in high intensity. With a workday of 480 min, these proportions correspond to 116 min, 357 min, and 7 min, respectively.

Only one worker had more than 10 min of high-intensity activity on ‘intervention’ workdays, and this worker was already sufficiently active during ‘control’ workdays.

### 3.7. Perceived Fatigue, Pain and Energy

**Figure 9:**
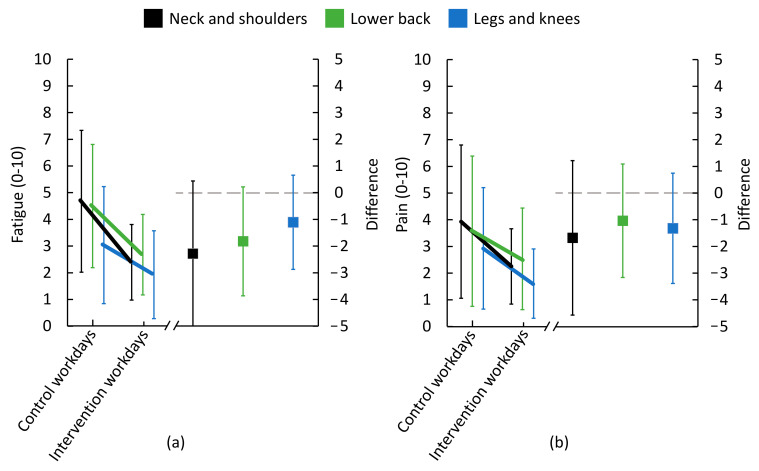
Perceived fatigue and pain immediately after work (*n* = 14). ‘Control’ workdays consisted of performing work as usual; ‘intervention’ workdays consisted of performing modified work. Ratings of (**a**) fatigue and (**b**) pain are shown on left y-axis. Difference between ratings of fatigue and pain after ‘control’ workdays and ‘intervention’ workdays are shown on right y-axis. Black, green and blue symbols represent neck and shoulders, lower back, and legs and knees, respectively. In the left part of the figure, lines illustrate group means, with bars showing standard deviation (SD). In the right part, squares represent differences between ‘control’ workdays and ‘intervention’ workdays. The grey dashed line indicates that ‘control’ and ‘intervention’ work did not differ; negative values indicate less fatigue/pain on ‘intervention’ workdays compared to ‘control’ workdays, i.e., a favorable change.

On average, workers reported less fatigue in neck and shoulders (M 2.4, SD 1.1, vs. M 4.7 SD, 2.7), lower back (M 2.7, SD 1.5, vs. M 4.5, SD 2.3) and legs and knees (M 1.9, SD 1.6, vs. M 3.0, SD 2.2) following ‘intervention’ workdays compared to ‘control’ workdays (Figure 9). Also, workers rated less pain in neck and shoulders (M 2.3, SD 1.4, vs. M 3.9, SD 2.9), lower back (M 2.5, SD 1.9, vs. M 3.6, SD 2.8), and legs and knees (M 1.6, SD 1.3, vs. M 2.9, SD 2.3) following ‘intervention’ workdays compared to ‘control’ days (Figure 9).

**Figure 10:**
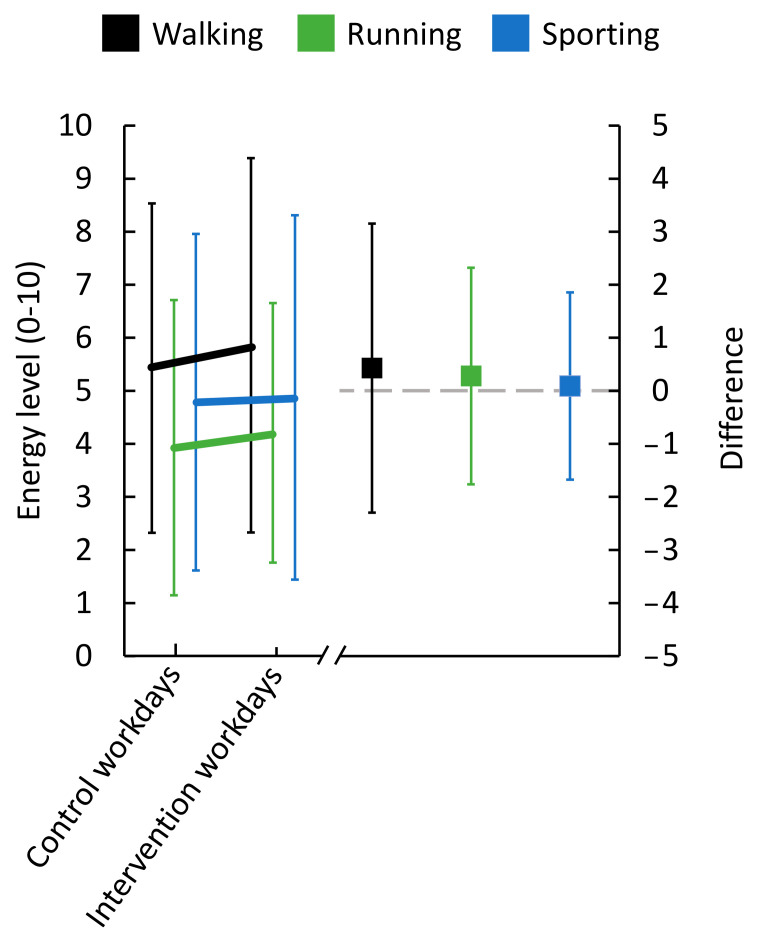
Perceived energy to perform 30 min of walking, running or undertaking sport immediately after work (*n* = 14). ‘Control’ work consisted of performing work as usual; ‘intervention’ workdays consisted of performing modified work. Ratings of energy level are shown on the left y-axis. Difference between ratings after ‘control’ workdays and ‘intervention’ workdays are shown on the right y-axis. Black, green, and blue symbols represent walking, running and undertaking sport, respectively. In the left part of the figure, lines illustrate group means, with bars showing standard deviation (SD). In the right part, squares represent differences between ‘control’ workdays and ‘intervention’ workdays. The grey dashed line indicates that ‘control’ and ‘intervention’ work did not differ; positive values indicate a higher energy level on ‘intervention’ workdays compared to ‘control’ workdays, i.e., a favorable change.

On average, workers rated more perceived energy for walking (M 5.9, SD 3.5, vs. M 5.4, SD 3.1), running (M 4.2, SD 2.4, vs. M 3.9, SD 2.8) and doing sports (M 4.9, SD 3.4, vs. M 4.8, SD 3.2), following ‘intervention’ workdays compared to ‘control’ workdays (Figure 10).

## 4. Discussion

The findings from this feasibility study suggest that it is, indeed, feasible to develop and implement modifications to productive work according to the Goldilocks Work Principle among industrial workers. When workers performed modified work, developed by themselves, their physical behavior changed towards the two stated goals for ‘intervention’ workdays, i.e., an equal proportion of time sitting, standing and being physically active, and at least 10 min in high intensity physical activity at ≥60%HRR. However, the changes did not fully reach these goals. Despite this, workers experienced less fatigue, less pain and a higher energy level following modified ‘intervention’ workdays compared to unmodified ‘control’ days.

### 4.1. Development and Implementation of ‘Just Right’ Modifications as Part of Productive Work

The workers developed five modifications of productive work at the workshops preceding implementation. Eventually, three of these were successfully implemented, which demonstrates the usefulness of the four steps procedure (cf. Figure 1). However, the failure to implement two of the five modifications also shows the importance of testing the feasibility of intended modifications in real productive work before large-scale implementation.

Workers reported why they did not perform the two unsuccessful modifications, i.e., ‘sitting folding cardboard’ and ‘sitting labelling task’. Several workers stated that when they had performed ‘sitting folding cardboard’ for 1 to 1.5 h, that would sufficiently supply the work team’s demand for folded cardboard for the entire day, which would preclude other workers from performing that task. As for the ‘sitting labelling task’, some workers stated that the chair offered felt uncomfortable, and some stated that it placed excessive strain on the upper extremity and/or lower back. Therefore, many of the workers did not perform this modification as planned.

### 4.2. Changes in the Distribution of Physical Behaviors

Three of the modifications of productive work aimed at increasing time sitting. However, on average only a minor increase was observed, i.e., 8 more minutes of sitting during the whole workday. This could be due to the aforementioned failures expressed by many of the workers regarding the modifications ‘sitting folding cardboard’ and ‘sitting labelling task’. Thus, the increase in average time sitting was likely primarily due to workers performing the ‘sitting meeting task’ as planned.

Although most of the workers increased their time being active on ‘intervention’ workdays compared to ‘control’ workdays, the average increase of only 9 min suggested that the modification that particularly aimed at a substantial increase in active time, i.e., ‘active moving task’, was not successful. This could be due to this task not being required much in production, which would lead to a limited time spent in this task, and thus being active, for all workers. Another explanation could be that workers did not increase their time being active while performing this modified task because they could perform the modification mainly standing, thus not being active.

### 4.3. Changes in Intensity

On average, the workers moved towards a ‘just right’ distribution of the three physical intensities; more time at low intensity (+18 min), less time with moderate intensity (−21 min), and more time at high intensity (+3 min) during ‘intervention’ workdays compared to ‘control’ workdays.

Three workers went from spending almost no time with high intensity during ‘control’ workdays to accumulating about five minutes of high intensity during ‘intervention’ workdays. As a general result, the developed modifications were not successful in leading to sufficient time at a high intensity, defined a-priori to be at least 10 min during worktime per workday. However, even though achieving five minutes with high intensity did not reach this goal, accumulating five minutes during worktime every workday could contribute to reach the weekly recommendations of 75 min of high intensity physical activity for adults [43].

### 4.4. Changes in Fatigue, Pain and Energy Level

We found a tendency towards workers being less fatigued, having less pain and reporting higher perceived energy level following ‘intervention’ workdays compared to ‘control’ workdays. The changes in physical behavior could have contributed to these positive changes. The workers were often standing for more than half of the workday, and even small increases in time sitting (+8 min) and some reduction of standing (−17 min) during ‘intervention’ workdays compared to ‘control’ workdays could have had effects [9,10,23,44]. Increased sitting may be reflected in the increased time spent at a low physical intensity (+18 min) during ‘intervention’ workdays compared to ‘control’ workdays, which is also a likely contributing explanation to the decreased level of fatigue at the end of the workday. Another explanation could be the increased variation resulting from an increased frequency of alternations between work tasks with different dominating physical behaviors on ‘intervention’ workdays compared to ‘control’ workdays. Increased variation—from low levels—is generally considered to reduce fatigue and pain [45,46]. Finally, the collaborative approach involving both management and many of the workers in the development of the modifications may have led to workers having inherently positive attitudes to the changes, thus reporting less fatigue and pain and higher energy level on ‘intervention’ workdays compared to ‘control’ workdays.

### 4.5. Practical Implications

The present study suggests that it is feasible in an industrial setting to develop modifications to productive work, based on the Goldilocks Work Principle. Furthermore, the study shows that implementing such modifications can change workers’ physical behavior and the intensity of activity towards intended goals, even if the present study showed these changes to be far from what was desired for a ‘just right’ workday. Thus, a paramount issue in future projects is to improve the success, both of developing effective modifications that can, actually, lead to the intended changes, and of implementing those modifications successfully. This could lead to larger changes in physical behavior and intensity than those observed in the present study if, for example, additional tailoring of modifications would be possible, based on workers feedback. Furthermore, the sustainability of changes needs to be evaluated, even if they appear feasible in the short term. The Goldilocks Work Principle argues that task modifications which do not impact negatively on productivity should be sustainable, but this needs to be evaluated. Also, the ability of such sustainable changes in behavior to positively change workers’ well-being, health and fitness needs to be examined.

Thus, in order to develop and implement modifications of productive industrial work based on the Goldilocks Work Principle, and examine their effects on workers’ health and fitness, larger scale controlled trials are encouraged. Effective modifications verified in a controlled trial design could offer a strategic and cost-effective way to promote industrial workers’ health and fitness, for the benefit of both workers, companies and society.

### 4.6. Strengths and Limitations

A strength of this study was the participatory approach used to develop and implement modifications to productive work. We involved workers, management, work environment representatives, and health and safety managers within the company in this whole process.

Another strength of this study was the use of wearable sensors to measure physical behaviors (i.e., sitting, standing, active) and intensity of physical activity (in terms of %HRR) during ‘control’ and ‘intervention’ workdays.

Some limitations should, however, be observed. First, the small sample size did not allow inferential statistics. Second, while we monitored alternations between work tasks on ‘intervention’ workdays compared to ‘control’ workdays, as a measure of the frequency aspect of variation, we did not explicitly evaluate the temporal pattern of physical behaviors, which is likely an important determinant of, e.g., fatigue and pain, even if the total duration of different behaviors is the same [47]. Future studies should include considerations of the duration of uninterrupted periods of sitting, standing and active in the operationalization of ‘just right’ work [48]. Third, data describing the use of time in, e.g., different physical behaviors or at different intensities of activity are ‘compositional’, in the sense that they are constrained (between 0% and 100%) and inherently dependent (change of time in one behavior is inevitably associated with change in at least one other behavior) [49]. Techniques, i.e., ‘Compositional Data Analysis’ (CoDA), have been developed to accommodate time use data [49], but we did not use CoDA in this study. We encourage future studies addressing time in physical behaviors and intensities to use CoDA. Fourth, the study design required researchers to engage with the workers during the workshops and consultations. This could have introduced bias in the results, in the sense that workers could have answered in favor of ‘intervention’ work in the questionnaires on fatigue, pain and energy following ‘control’ and ‘intervention’ workdays, to accommodate the researchers. Fourth, the Goldilocks Work Principle emphasizes that modifications to work must not compromise productivity. In the present study, we only assessed productivity in terms of the conviction of participants at the workshops that the developed modifications would not have a negative effect. We encourage future studies to collect more solid data addressing productivity effects as well as cost benefits of the intervention. Fifth, we collected self-reported data to assess whether participants were compliant in practicing the intended modifications to work on ‘intervention’ workdays. This approach relies on the participants’ knowledge of the modified work as well as their ability and willingness to report whether they did, in fact, perform the modification according to plan. We encourage future studies to collect observational data to assess compliance in performing modified work as planned. Sixth, the small sample size in the present study, i.e., less than 10 workers, could have introduced bias, both in terms of the sample not being representative for this group, and in terms of workers participating having a more positive attitude to the planned modifications than their colleagues in general. Thus, we encourage future studies to include a larger sample of workers and take measures to increase workers’ willingness to wear sensors. Finally, in the current study participants acted as their own controls (i.e., all participants practiced both ‘control’ and ‘intervention’ workdays). This design could result in physical behavior on ‘control’ workdays not truly reflecting behaviors during usual work. Therefore, we encourage future studies to have a separate group of workers acting as controls.

The Goldilocks Work Principle is not intended to address all challenges to health among industrial workers. Thus, additional interventions (e.g., focusing on diet or alcohol habits) may be justified for these workers. In future intervention studies, the long-term effects of Goldilocks changes may be compared in terms of the cost-effectiveness of other organizational and individual initiatives aiming at promoting health.

## 5. Conclusions

The present study suggests that it is feasible to involve workers and other stakeholders in developing and implementing modified work based on the Goldilocks Work Principle in an industrial setting. Physical behavior and the intensity of physical activity changed towards the intended ‘just right’ workday, with an equal distribution of physical behaviors (i.e., equal proportions of sitting, standing and active), and sufficient time in high intensity to improve fitness. However, the modified work was still quite far from these target outcomes. We observed, however, that workers experienced less fatigue, less pain and had higher energy levels after the ‘intervention’ workdays compared to ‘control’ workdays. Thus, the present feasibility study provides useful knowledge for further development of ‘just right’ industrial work and, eventually, larger scale controlled trials assessing the effectiveness of modifications of productive work aiming at changing physical behavior and intensity and their efficacy in promoting health and fitness among the workers.

## Figures and Tables

**Table 1:** Characteristics of workers participating at baseline (*n*=20).

Variables	*N*	%	Mean (SD)
Sex (female)	7	35.0	
Age (years)			44.8 (10.9)
Length of service in current job			
*3–11 months*	8	40.0	
*12–120 months*	9	45.0	
*>120 months*	3	15.0	
Self-rated productivity (0–10)			8.5 (1.3)
Self-rated physical exertion during work (0–10)			5.6 (2.0)
Self-rated pain (0–10)			
*Neck and shoulders*			3.1 (2.6)
*Lower back*			3.5 (2.9)
*Legs and knees*			2.1 (2.6)
Self-rated time spent in MVPA during work and leisure (hours/week)			11.9 (11.0)
Self-rated time spent in MVPA during work (hours/week)			5.8 (7.9)
Self-rated time spent performing VPA during work and leisure (hours/week)			2.1 (2.6)
Current smokers (yes)	5	25.0	
BMI (kg/m^2^)			27.4 (4.0)
Normal weight (i.e., 18.5–24.9)	7	35.0	
Overweight (i.e., ≥25)	9	45.0	
Obese (i.e., >30)	4	20.0	
Fat percentage (%)			27.4 (6.9)
Blood pressure (Systolic/diastolic, mmHg)			
*Normal blood pressure (i.e., <130/80)*	9	45.0	
*Elevated blood pressure (i.e., >130/80)*	5	25.0	
*Hypertension (i.e., >140/90)*	6	30.0	

Abbreviations: MVPA = Moderate to Vigorous Physical Activity; VPA = Vigorous Physical Activity; BMI = Body Mass Index; SD = Standard Deviation. *Italic* indicates a specification or subcategory of the header.

## Data Availability

The data presented in this study are available on request from the corresponding author. The data are not publicly available due to general data protection regulations (GDPR), and personal data ordinance (PDPO).

## Appendix A

**Table A1:** Physical behavior (*n* = 5) and intensity (*n* = 15) among workers in the initial survey of industrial work (step 1, cf. Figure 1).

Variables	Mean (SD)	Proportion of Work Time (%)
Physical behavior		
*Sitting*	128 (65) min	27
*Standing*	266 (76) min	56
*Active*	80 (33) min	17
Intensity		
*Low intensity (* *<25%HRR)*	98 (82) min	20
*Moderate intensity (* *≥25–<60%HRR)*	378 (79) min	79
*High intensity (* *≥60%HRR)*	5 (9) min	1

Sitting = sitting and lying; Standing = stationary standing, and standing with limited movement; Active = walking, running, stair climbing and cycling; HRR = heart rate reserve; SD = standard deviation. *Italic* indicates a specification or subcategory of the header.

## Appendix B

Step 1. We collected field observations and had dialogues at the workplace revealing that the eight-hour workday was primarily spent at one of three workstations, and included two rest breaks of 30 min each. The workers were organized in three teams, two teams alternating weekly between day shifts (7.00–15.00) and evening shifts (15.00–23.00), and one team exclusively performing night shifts (23.00–7.00). The three main work tasks were: (1) a ‘moving task’ where workers manually relocated bottle cases from one pallet to another, (2) an ‘assemble quarter pallet’ task where workers assembled a quarter pallet with bottles, cardboard and foil, and (3) a ‘labelling task’ where workers labeled bottles with a deposit mark. Typically, tasks at each workstation were performed once daily for approximately two hours in a standing position. The ‘moving task’ involved moving, walking, lifting and labeling cases. ‘Assemble quarter pallet’ involved moving, walking and stacking cardboard and bottles. The ‘labelling task’ involved stationary standing, labelling bottles and moving cases of bottles from a table to a pallet.

Step 2. We collected measurements using wearable sensors and showed that workers were, on average, sitting for 2 h, standing for about 4.5 h, and were active (i.e., walking, running, stair climbing, or cycling) for about 1 h (Table A1). Heart rate measurements showed that workers spent 1.5 h with low intensity (<25%heart rate reserve, HRR), more than 6 h with moderate intensity (≥25 to <60%HRR), and about 5 min at high intensity (≥60%HRR).

Step 3. With the aim of ensuring workers approach a ‘just right’ distribution of physical behaviors during work, we decided to aim at (1) increasing time sitting and time active, and (2) including periods with high intensity. The increase in time spent sitting could be achieved by modifying low-intensity standing work tasks to, instead, be performed sitting, and the increase in active time could be obtained by modifying more intense standing work to be performed while moving around. The increase in periods with high intensity could be achieved by modifying physically demanding work tasks to be performed at an even faster pace.

Step 4. At workshops involving workers and other company stakeholders, five modifications of work were developed, with the intention to have workers moving towards a ‘just right’ distribution of physical behaviors, and an increase in time with high intensity. The five modifications were termed, (1) ‘Active moving task’, (2) ‘Sitting folding cardboard’, (3) ‘Sitting labelling task’, (4) ‘Sitting meeting task’, and (5) ‘Increase alternations between tasks’. These tasks were then implemented and evaluated in this study.

## Appendix C

**Table A2:** Two-hour workshop performed at the workplace.

Activity	Purpose	Method and Process
Introduction	To establish a common understanding of the project aim and why it is important/meaningful	Workers are introduced to the Goldilocks Work Principle.
Current physical behavior and intensity	To help workers’ understand their own physical activity behavior and workload during work and what is missing.	Workers are presented with their own ambulatory measurements of physical activity on a group level.
Redesign work	To support workers’ redesigning current work tasks to move workers towards ‘just right’ distribution of physical activity behavior. To support workers to select the redesigns with the best potential to change targeted physical behavior during work.	In groups of 3–5, workers develop as many redesigns of current work tasks they can think of. Subsequently, all workers’ collectively select which redesigned work tasks that has the best potential to change work behavior towards ‘just right’. Each idea for modification was assessed by all workshop participants in terms of five criteria: (1) Would it contribute to changing physical behavior and/or intensity towards a ‘just right’ distribution? (2) Is it part of the productive work? (3) Are there facilitating factors supporting implementation of this modification (e.g., is it fun to do)? (4) Are there barriers to the implementation of this modification (e.g., is it expensive, impractical)? (5) How would it affect overall productivity? All modifications were scored in each category as either positive, neutral, or negative.
Planning implementation	To support workers to plan the implementation of the redesigned work tasks.	Process of selecting key persons who helps to implement the redesigned work tasks. Questions asked (for each redesign): What should the workers do? What should the management do? What should the work environment representative do?
Evaluation of the workshop	To evaluate the workers’ motivation for participation and their perception of the workshop.	All participating workers fill out an evaluation questionnaire

## Appendix D

**Table A3:** Workers’ opinion about of the workshop according to questionnaires answered just after the two workshops, one for the dayshift and one for the evening shift (*n* = 16) *.

Variables	*N*	%	Mean (SD)
Do you look forward to participating in this project?			
*To a low degree*	1	6	
*Somewhat*	1	6	
*To a large degree*	14	88	
Are you motivated to participate in this workshop?			
*To a low degree*	0	0	
*Somewhat*	0	0	
*To a large degree*	16	100	
Has the overall content of the workshop been easy to comprehend?			
*To a low degree*	0	0	
*Somewhat*	2	12	
*To a large degree*	14	88	
Did you understand the presentation of baseline measurements (i.e., physical behavior and intensity)?			
*To a low degree*	0	0	
*Somewhat*	2	12	
*To a large degree*	14	88	
Did the collaboration with your team work well in this workshop?			
*To a low degree*	0	0	
*Somewhat*	3	19	
*To a large degree*	13	81	
Do you think that the results of the baseline measurements is something that you and your team can use in the future?			
*To a low degree*	1	6	
*Somewhat*	4	24	
*To a large degree*	11	70	
Did your team leader support you and your team in the workshop?			
*To a low degree*	0	0	
*Somewhat*	3	18	
*To a large degree*	13	82	
Did you benefit overall from participating in this workshop?			
*To a low degree*	1	6	
*Somewhat*	2	12	
*To a large degree*	13	81	
Do you feel informed about what will happen following the workshop?			
*To a low degree*	0	0	
*Somewhat*	2	12	
*To a large degree*	14	88	
Are you motivated to participate in the modifications and changes at your workplace following this workshop?			
*To a low degree*	0	0	
*Somewhat*	2	12	
*To a large degree*	14	88	
Rate the performance of the course leader (0–5)			4.8 (0.4)

* 14 workers participated in total, as well as the team leader and work environment representative (WER). Team leader and WER only filled out the questionnaire following the day shift workshop. *Italic* indicates a specification or subcategory of the header.
